# A Mechanically Reinforced Super Bone Glue Makes a Leap in Hard Tissue Strong Adhesion and Augmented Bone Regeneration

**DOI:** 10.1002/advs.202206450

**Published:** 2023-01-25

**Authors:** Shanshan Hu, Shan Wang, Qingqing He, Dize Li, Liangjing Xin, Chuanhang Xu, Xingyu Zhu, Li Mei, Richard D. Cannon, Ping Ji, Han Tang, Tao Chen

**Affiliations:** ^1^ Stomatological Hospital of Chongqing Medical University Chongqing Key Laboratory of Oral Diseases and Biomedical Sciences Chongqing Municipal Key Laboratory of Oral Biomedical Engineering of Higher Education Chongqing 401147 P. R. China; ^2^ Department of Oral Sciences Sir John Walsh Research Institute Faculty of Dentistry University of Otago, Dunedin Dunedin 9054 New Zealand

**Keywords:** bone fracture, L‐dopa amino acid, mussel, osteogenesis, zeolitic imidazolate framework‐8

## Abstract

Existing bone tissue engineering strategies aim to achieve minimize surgical trauma, stabilize the injured area, and establish a dynamic osteogenic microenvironment. The cutting‐edge bone glue developed in this study satisfies these criteria. Inspired by the excellent adhesive properties of mussels, herein, a super osteogenic glue (L‐DPZ) that integrates poly(vinyl alcohol), L‐dopa amino acid, and zeolitic imidazolate framework‐8 characterized by catechol–metal coordination is used to successfully adhere to hard tissue with a maximum adhesive strength of 10 MPa, which is much higher than those of commercial and previously reported bone glues. The stable hard tissue adhesion also enables it to adhere strongly to luxated or broken teeth, Bio‐Oss (a typical bone graft material), and splice fragments from comminuted fractures of the rabbit femur. Then, it is testified that the L‐DPZ hydrogels exhibit satisfactory biocompatibility, stable degradability, and osteogenic ability in vitro. Moreover, the ability to anchor Bio‐Oss and sustained osteogenesis of L‐DPZ result in satisfactory healing in calvarial bone defect models in rabbits, as observed by increased bone thickness and the ingrowth of new bone tissue. These results are expected to demonstrate solutions to clinical dilemmas such as comminuted bone fracture fixation, bone defect reconstruction, and teeth dislocation replantation.

## Graphical abstract

An ultra‐high strength orthopedic adhesive to splice bone fragments and adhere bone grafts is reported, whilst also guiding subsequent bone healing. This adhesive integrates poly(vinyl alcohol), L‐dopa amino acid, and the zeolitic imidazolate framework‐8, which opens up a promising avenue for many clinical dilemmas such as comminuted fracture fixation and bone defect reconstruction.

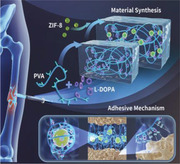

## 1 Introduction

Treating bone fractures caused by trauma, tumors, and orthopedic surgery remains one of the most common clinical challenges; it has impacted the health of tens of millions of people worldwide.^[^ ^1^ ^]^ Although traditional technologies, such as plates, screws, and pins, are commonly adopted in the clinical setting, they often cause foreign‐body reactions that require additional surgery to be resolved.^[^ ^2^ ^]^ This is particularly true in the case of severe and highly comminuted bone fractures or massive hemorrhages, wherein the surgical splice of small fragments accompanied by continuous bleeding becomes extremely difficult and time‐consuming and can cause serious complications or even become life‐threatening.^[^ ^1^ ^]^ In such circumstances, instant hemostasis and strong fixation of the intricate bone fragments are pivotal. Bone glues represent a promising alternative for bone fracture fixation in this context.^[^ ^3^ ^]^

Bone glues, including cyanoacrylate (CA), poly(methyl methacrylate) (PMMA), and calcium phosphate cement (CPC), are commonly used clinically for bone repair.^[^ ^2^, ^4^ ^]^ However, such adhesives still have several limitations. For example, CA exhibits poor biodegradability and biocompatibility,^[^ ^3^, ^4^ ^]^ while PMMA lacks intrinsic adhesion properties, induces significant thermal necrosis, and leaks toxic monomers.^[^ ^2^, ^4^ ^]^ In addition, the clinical application of CPC is limited by its poor mechanical properties and facile collapse in a wet environment.^[^ ^2^, ^4^ ^]^ Therefore, various types of hydrogel‐based bone glues have been explored to resolve the above‐mentioned deficiencies of existing products, with example systems including fibrin adhesives, polysaccharides, and silkworm mucin.^[^ ^5^ ^]^ Although these hydrogel‐based bone glue may exhibit satisfactory biosafety and biodegradability profiles, they still lack sufficient adhesion to bone tissues, especially in wet environments with continuous bleeding. In particular, for bones where stable fixation is necessary (e.g., in the femur), strong adhesion strength and mechanical stability in the wet bone environment are highly desirable.^[^ ^4^, ^6^ ^]^ Furthermore, the ideal bone glue should be bioresorbable and be biodegraded into biocompatible products concomitantly with the formation of new bone, without requiring additional surgery to remove the fixative.^[^ ^4^ ^]^ Moreover, to accelerate structural restoration and healing in severe bone fractures, novel bone glues should also exhibit osteogenic properties.^[^ ^2^, ^4^ ^]^ It is therefore of vital importance to develop a product with ultra‐strong wet adhesiveness, an appropriate degradation rate, and a satisfactory osteogenic activity.

Inspired by the excellent adhesive capability of marine mussels in wet environments, mussel adhesive proteins (MAPs) have been identified as a promising model for next‐generation bio‐inspired adhesives.^[^ ^7^ ^]^ Within MAPs, L‐dopa amino acid (L‐DOPA), a modified form of tyrosine, has attracted widespread attention due to its excellent biocompatibility, water resistance, and strong adhesion properties.^[^ ^7^, ^8^ ^]^ In a structural context, L‐DOPA‐containing MAPs possess a large number of functional catechol moieties, which can form dynamic noncovalent bonds with various substrates through hydrogen bonding, in addition to covalent bonds via Schiff base or Michael addition reactions.^[^ ^7^, ^9^ ^]^ In addition, L‐DOPA exhibits a strong metal ion chelating capacity, and the resulting catechol–metal coordination bonds result in improved cohesive interactions and mechanical properties, which have stimulated the development of mussel‐inspired metallopolymer materials.^[^ ^7^, ^10^ ^]^ We, therefore, hypothesized that an L‐DOPA‐modified hydrogel could be an ideal bone glue candidate for application to severe and highly comminuted bone fractures under massive hemorrhage conditions.

The inability of L‐DOPA‐modified nanomaterials to guide bone regeneration is currently a limitation of these materials.^[^ ^1^, ^11^ ^]^ Therefore, it is necessary to develop modified strategies aimed at strong bone adhesion and satisfactory osteogenesis. In this context, metal–organic frameworks (MOFs), which are composed of metal ions/clusters and organic ligands, have attracted substantial attention in recent years due to their ultrahigh surface areas, their high and uniform porosities, and their good biodegradabilities.^[^ ^12^ ^]^ For example, zeolitic imidazolate framework‐8 (ZIF‐8), an important subclass of MOFs, has emerged as a promising reagent in the tissue engineering field.^[^ ^13^ ^]^ Indeed, our previous studies have demonstrated that the controlled and sustained release of Zn^2+^ from ZIF‐8 shows promise for use in bone regeneration.^[^ ^1^, ^14^ ^]^ Moreover, ZIF‐8 also has the potential to enhance the mechanical strengths of hydrogels.^[^ ^1b^ ^]^

**Figure 1:**
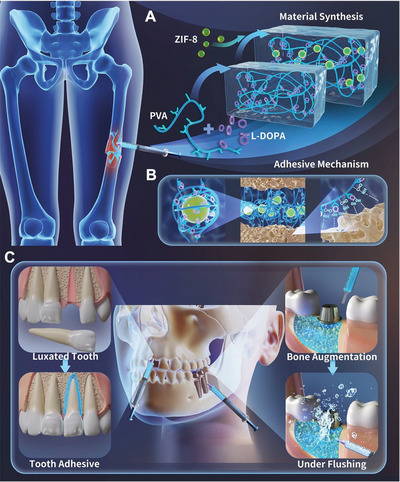
Schematic diagram showing the design principle of a mechanically reinforced super bone glue hydrogel (A), as well as its adhesive mechanism (B) and multiple clinical application scenarios (C).

In this study, in order to construct a biomimetic bone glue with an excellent biosafety profile, superglue‐like adhesion strength, and satisfactory osteogenic properties in relevant biological environments, the biocompatible poly(vinyl alcohol) (PVA) polymer was used as long chain structure and ZIF‐8 nanoparticles (ZIF‐8 NPs) were used to functionalize the bio‐adhesive L‐DOPA‐PVA (L‐DP) to form a highly integrated metal–catecholamine coordination structure (**Figure** **1**A). Then, the synthesis, injectability, and self‐healing ability of L‐DOPA‐PVA‐ZIF‐8 (L‐DPZ) hydrogel was first verified. The integration of ZIF‐8 and L‐DP at a nanoscale level increased mechanical strength enabling it to confer strong adhesion with bovine bone tissue, ex vivo, with a lap shear strength of 10 MPa, and splice bone fragments in a rabbit femoral comminuted fracture model, in vivo. As shown in Figure 1B, the strong adhesion could be mainly attributed to the following mechanisms: 1) Abundant free catechol groups of L‐DOPA. 2) Chemical interactions between catechol–quinone groups and amine groups through Schiff‐base reactions. 3) The intermolecular association between polymer and ZIF‐8. L‐DPZ also performed well when applied to hard tissues in the maxillofacial region (Figure 1C, left panel), like fractured and luxated teeth. To further test its potential as an aid to bone grafting, commercial Bio‐Oss granules were mixed with the L‐DPZ hydrogel to form a hydrogel‐granule composite. The injectability, compressive strength, and bending behavior of L‐DPZ‐Bio‐Oss composites, as well as the fixation retention of L‐DPZ to Bio‐Oss in the implant exposed area (Figure 1C, right panel) and extraction socket, were investigated. Finally, when the L‐DPZ‐Bio‐Oss composite was used in a rabbit calvarial bone defect model, active bone regeneration and considerable bone augmentation were confirmed. We believe that this ingenious bone glue shows great potential for the treatment of severe bone fractures and bone defects and might provide new insights into the generation of more innovative and cutting‐edge bone glues in the future.

## 2 Results and Discussion

### 2.1 Synthesis and Characterization of the L‐DPZ Hydrogel

**Figure 2:**
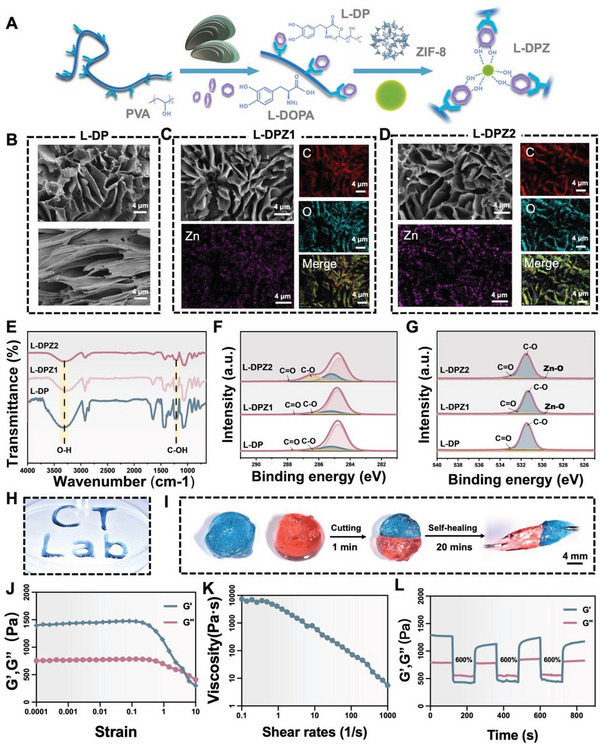
Structural and mechanical properties of the L‐DPZ hydrogels. A) Schematic illustration of the synthesis of the L‐DPZ hydrogels. B) SEM images of the surface and cross‐section of the L‐DP hydrogel. SEM and EDS images of the C) L‐DPZ1 and D) L‐DPZ2 hydrogels. E) FTIR spectra of the L‐DP, L‐DPZ1, and L‐DPZ2 hydrogels. F) C1s and G) O1s high‐resolution XPS spectra of the L‐DP, L‐DPZ1, and L‐DPZ2 hydrogels, respectively. H) Photographic image showing the injectability of the L‐DPZ2 hydrogel, wherein a blue dye was used to stain the hydrogel sample for visibility purposes. I) Photographic images demonstrating the self‐healing behavior of the L‐DPZ hydrogel. J) Variation in the storage modulus (*G*′) and the loss modulus (*G*′′) of the L‐DPZ2 hydrogel, as determined using the strain amplitude sweep test (0.01–1000%). K) Variation in the viscosity of the L‐DPZ2 hydrogel, as determined using the shear‐thinning test. L) Variations in *G*′ and *G*′′ for the L‐DPZ2 hydrogel, as observed during three alternate step‐strain tests.

The L‐DPZ hydrogel fabrication procedure is illustrated in **Figure** **2**A. Detailed characterization of the starting L‐DP polymer is provided in our previous study.^[^ ^9^ ^]^ Thus, the micro‐morphologies of the L‐DP, L‐DPZ1, and L‐DPZ2 hydrogels (named according to the mass ratio of L‐DP to ZIF‐8 is 100/1 or 100/2; please refer to the Experimental Section, Supporting Information, for more details) were analyzed through scanning electron microscopy (SEM). As depicted in Figure 2B–D, all hydrogels exhibited a porous 3D structure. However, with the addition of ZIF‐8, a more regular pore structure with fewer discontinuities was obtained. Analysis by energy dispersive spectroscopy (EDS) showed that the Zn^2+^ ions were evenly distributed within the hydrogel, thereby confirming the successful incorporation of ZIF‐8. The L‐DP and L‐DPZ hydrogels were then characterized by Fourier transform infrared (FTIR) spectroscopy (Figure 2E), wherein the strong and broad absorption band corresponding to the hydroxyl groups in L‐DP (3343 cm^−1^) decreased in intensity and shifted to 3243 and 3287 cm^−1^ in L‐DPZ1 and L‐DPZ2, respectively.^[^ ^15^ ^]^ In addition, the intensity of the phenolic C—O—H vibration peak of the catechol groups (1200–1240 cm^−1^) was also reduced to a large extent in both L‐DPZ1 and L‐DPZ2. These results suggest that physical crosslinking and catechol–Zn^2+^ coordination were both present in this system.^[^ ^15^ ^]^ The structures of L‐DPZ1 and L‐DPZ2 were further verified by X‐ray photoelectron spectroscopy (XPS).^[^ ^16^ ^]^ More specifically, the high‐resolution C1s and O1s spectra presented in Figure 2F,G show enhancement of the C—O, C—N, and C=O signals at 286.5, 285.2, and 532.9 eV, respectively, in addition to the appearance of a Zn—O signal at 529.7 eV for both L‐DPZ1 and L‐DPZ2. These results, therefore, confirm the presence of interactions between ZIF‐8 and L‐DP.

The injectability and self‐healing behavior of the L‐DPZ hydrogel system was then assessed using the L‐DPZ2 hydrogel as an example. As shown in Figure 2H, the L‐DPZ2 hydrogel was easily dispensed using a syringe, allowing letters to be written using the hydrogel. In addition, to assess the self‐healing properties of the hydrogel, two disks of L‐DPZ2 (stained blue and red) were cut in half and reassembled after exchanging the red and blue halves. Instant reconnection of the two semicircles was achieved in 1 min. After reconnection for 20 min, it was observed that the new disk could withstand a strong stretch perpendicular to the healed interface, demonstrating the effective self‐healing property of the L‐DPZ hydrogel (Figure 2I). It was therefore considered that the self‐healing capacity of the L‐DPZ hydrogel is not only due to *π*–*π* and hydrogen bonding between the catechol groups, but from coordination between the catechol groups and the Zn^2+^ ions present in ZIF‐8.^[^ ^17^ ^]^ Furthermore, rheological measurements were undertaken to further evaluate the self‐healing performance and mechanical properties of the hydrogel.^[^ ^17^, ^18^ ^]^ Thus, the viscoelastic behavior of the L‐DPZ hydrogel was first examined using the strain amplitude sweep test, and it was found that the storage modulus (*G*′) and the loss modulus (*G*′′) of the hydrogel remained almost constant under low strains, indicating that the L‐DPZ hydrogel exhibited a dominant elastic property (Figure 2J). Subsequently, the shear‐thinning behavior of the L‐DPZ hydrogel was evaluated, and it was found that the viscosity of the hydrogel decreased substantially from 7391 to 5.55 Pa s when the shear rate was increased from 0.1 to 1000 s^−1^ (Figure 2K). This behavior imparts the hydrogel with good injectability, which is a necessary requirement for coating irregular bone fragments during use as a bone glue. At high levels of strain, the *G*′ and *G*′′ values decreased dramatically and intersected at ≈600%, indicating that the hydrogel existed between the solid and fluid states close to its breaking point while behaving as a fluid at high strain values.^[^ ^17^, ^18^ ^]^ Continuous cyclic strain measurements were then carried out to evaluate the rheological recoverability of the L‐DPZ hydrogel. As shown in Figure 2L, at 600% strain, the *G*′ dropped drastically and became lower than *G*′′. However, once the stain was returned to 1%, *G*′ recovered immediately to its initial state, even after three consecutive cycles. This rapid sol–gel transition performance of the L‐DPZ hydrogel thereby confirmed the excellent self‐healing capacity of the hydrogel.

### 2.2 Ex Vivo Superstrong Adhesion Strength of L‐DPZ Hydrogel

**Figure 3:**
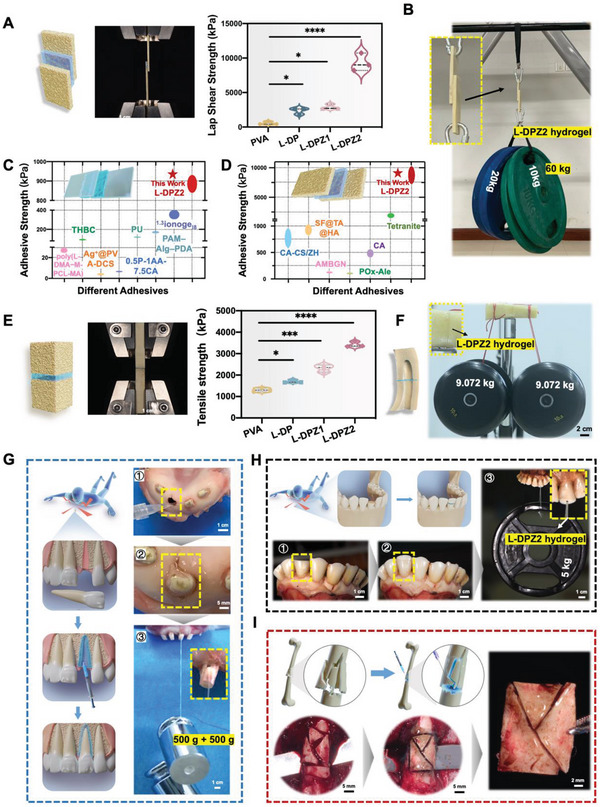
Characterization of the adhesive strength of the hydrogels toward different interfaces. A) Schematic representation of the lap shear adhesion test and the lap shear adhesion values for the different hydrogels on the bovine bone (*n* = 3). B) Lifting of 60 kg dumbbells using the bovine bone plates bonded with the L‐DPZ2 hydrogel over an area of 4 cm × 3 cm. C) Comparison of the adhesion strength of the L‐DPZ2 hydrogel with those of other adhesives on a glass substrate. D) Comparison of the adhesion strength of the L‐DPZ2 hydrogel with other adhesives and CA on a bovine bone substrate. E) Schematic representation of the tensile adhesion test and tensile strength values for the different hydrogels on bovine femur bone (*n* = 3). F) Lifting of 9.07 kg dumbbells using the bonded bovine femur bone fragments. G–I) Schematic representation and photographic images illustrating the use of the L‐DPZ2 hydrogel to repair a luxated tooth (G), a broken tooth (H), and the comminuted bone of a rabbit femur in vivo (I). *p < 0.05, **p < 0.01, ***p < 0.001, ****p < 0.0001, ns, not significant. Data were analyzed using one‐way ANOVA with Tukey's post hoc analysis.

To assess the potential of the L‐DPZ hydrogel for application as a bone glue in different clinical scenarios, various ex vivo experiments were undertaken. First, a lap shear test was used to measure the adhesion strength to various substrates, including polytetrafluoroethylene (PTFE), glass, and bovine bone, using a universal testing machine (**Figure** **3**A and Figure S1A,B, Supporting Information).^[^ ^19^ ^]^ The results demonstrated that the addition of L‐DOPA increased the adhesive strength, while the addition of ZIF‐8 further boosted the adhesion strength with all tested surfaces. As a result, the strongest adhesion performance was obtained using the L‐DPZ2 hydrogel. More specifically, the lap shear adhesion strength of the L‐DPZ2 hydrogel (9.31 ± 1.29 MPa) on the bovine bone surface was ≈17, 3.92, and 3.20 times higher than those of the PVA, L‐DP, and L‐DPZ1 hydrogels, respectively. Even in the case of the PTFE surface, which possesses ultralow surface energy, the shear strength of the L‐DPZ2 hydrogel reached 0.57 ± 0.03 MPa, which is significantly higher than the values given for previously reported adhesives.^[^ ^19b^ ^]^ These results indicate that the presence of catechol groups contributes to the enhanced adhesion strength of the hydrogel, while the incorporation of ZIF‐8 into the L‐DP hydrogel played an important role in improving the adhesion behavior due to coordination between the L‐DOPA catechol groups and the Zn^2+^ ions in ZIF‐8. Considering the excellent adhesion performance of the L‐DPZ2 hydrogel on bone substrates, further experiments were carried out, wherein this hydrogel was applied to different surfaces. For example, as shown in Figure 3B and Movie S1, Supporting Information, two bovine bone plates with a glued overlap area of 4 cm × 3 cm, which is a significantly smaller area than those examined previously,^[^ ^20^ ^]^ were bonded together using the L‐DPZ2 hydrogel, and it was found that the glued structure could be used to easily hang several dumbbells with a total weight of 60 kg. In addition, when two bonded plates were used to link a swing on two sides, one adult man (100 kg) or two adult women (120 kg total) were able to use the swing without detachment (Figure S1C and Movie S1, Supporting Information). Thus, based on the excellent lap shear strength of the L‐DPZ2 hydrogel, its adhesion strength was compared with those of other adhesives reported in the literature. As shown in Figure 3C,D, the average adhesion strengths of the L‐DPZ2 hydrogel with the glass (Figure 3C)^[^ ^21^ ^]^ and bovine bone (Figure 3D)^[^ ^1^, ^2^, ^4^, ^22^ ^]^ substrates were significantly higher than those of the reported adhesives and the commercially available bone glue cyanoacrylate (CA). Subsequently, the adhesiveness of the L‐DPZ2 hydrogel was further assessed by means of a tensile adhesion test, and as a result, the tensile strength was calculated to be 3.42 ± 0.13 MPa; this value is significantly higher than those of the PVA, L‐DP, and L‐DPZ1 systems, which were 1.30 ± 0.09, 1.70 ± 0.07, and 2.32 ± 0.17 MPa, respectively (Figure 3E). Moreover, adhesion of the L‐DPZ2 hydrogel to two pieces of broken femur bone was demonstrated, and the firmly glued bone was successfully used to support two dumbbells, each weighing 9.07 kg (Figure 3F).

Teeth are the hardest tissue in the human body, and like bones, they are frequently fractured or luxated as a result of trauma.^[^ ^23^ ^]^ Indeed, it is extremely difficult to restore luxated teeth since the firm adhesion of the teeth into the alveolar sockets, which contain both soft and hard tissue, is challenging.^[^ ^23^, ^24^ ^]^ To test whether the L‐DPZ hydrogel is suitable for gluing teeth, a broken piece of the bovine anterior tooth was employed, and after firm bonding with the glue, the tooth was able to support a dumbbell weighing 5 kg (Figure 3H). When the weight of the dumbbell was increased, the tooth broke, while the adhered surface remained intact (Figure S1D, Supporting Information), thereby demonstrating the excellent adhesiveness of the L‐DPZ2 hydrogel to the tooth surface. In addition, as presented in Figure 3G, the L‐DPZ2 hydrogel successfully glued a luxated tooth into the alveolar socket of a pig, and the resulting tooth was able to support two weights (1 kg in total, see Movie S2, Supporting Information).

The adhesive performances of the L‐DPZ2 hydrogel in aqueous environments were then evaluated both in vitro and ex vivo. For this purpose, the head of a fresh pig femur was cut and the L‐DPZ2 hydrogel was injected onto the cut surface. After rinsing the hydrogel for 1 min under fast‐running water, it remained tightly adhered to the bone surface (Figure S1E and Movie S3, Supporting Information). Furthermore, to simulate the clinical situation, the adhesiveness of the L‐DPZ hydrogel was tested ex vivo in the presence of blood. Using an in vivo comminuted fracture model on a rabbit femur (Figure 3I), the hydrogel was injected onto the fracture surfaces of the bone fragments, and the bone pieces were connected immediately. To further quantify the instant wet adhesive ability of the hydrogel, a lap shear experiment was conducted (Movie S4, Supporting Information), and the instant wet adhesive strength of L‐DPZ2 reached 453.5 ± 64.6 kPa (Figure S1F, Supporting Information), which indicates the feasibility of clinical application of the L‐DPZ hydrogel as an effective bone glue and allows surgeons sufficient time to perform the operation before the hydrogel was fully cured.

It was also demonstrated that this hydrogel exhibited an incredibly strong adhesion strength with a variety of surfaces including PTFE, glass, bone, and tooth enamel. For example, in the case of bovine bone, the highest adhesion strength was >10 MPa (Figure 3A), which was significantly higher than the values reported in the literature for other adhesives^[^ ^1^, ^2^, ^4^, ^22^ ^]^ and for the commercial bone glue CA. This strong adhesiveness observed for the L‐DPZ hydrogel was therefore attributed to several mechanisms (Figure 1B): 1) Physical interactions, such as *π*–*π* stacking and hydrogen bonding interactions caused by the presence of abundant free catechol groups on L‐DOPA;^[^ ^17a^ ^]^ 2) Chemical interactions between the catechol–quinone groups and the amine or thiol groups by means of Michael addition and Schiff‐base reactions;^[^ ^9^, ^17^ ^]^ 3) Intramolecular crosslinking between the polymer chains and intermolecular associations between the polymer and ZIF‐8;^[^ ^17^, ^20^, ^25^ ^]^ and 4) Coordination bonds between the phenolic moieties of L‐DOPA and the Zn^2+^ ions originating from ZIF‐8.^[^ ^2a^ ^]^ As a result of the above interactions, the L‐DPZ hydrogel was able to achieve quick and firm fixation to hard tissues, and would therefore be expected to generate innovative solutions for achieving hard tissue adhesion, especially in highly comminuted bone fractures and severe dental traumas.

### 2.3 Ex Vivo Xenogenic Bone Substitute Fixation Capability of the L‐DPZ Hydrogel

**Figure 4:**
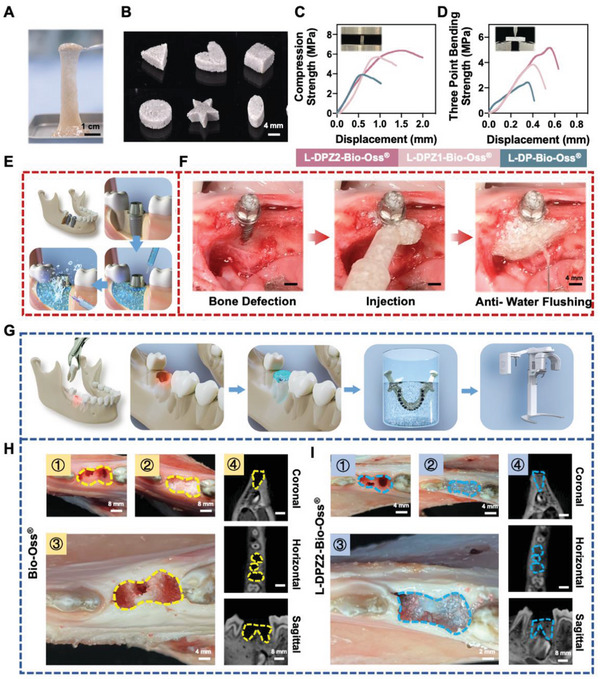
Characterization on stable fixation of L‐DPZ2‐Bio‐Oss composite. A) Macroscopic photograph of the formation of the hydrogel‐Bio‐Oss composite. B) Photograph of the hydrogel‐Bio‐Oss composite reshaped into different shapes. C) Representative stress‐displacement curves for different hydrogel‐Bio‐Oss composites undergoing the uniaxial compression test. D) Representative stress‐displacement curves for different hydrogel‐Bio‐Oss composites undergoing the three‐point bending test. E) Schematic and F) photographs showing the use of L‐DPZ2 hydrogel with water flushing in an ex vivo alveolar bone model using a Bama Miniature pig. G) Schematic illustrating the process of evaluating the long‐term stability of the L‐DPZ2 hydrogel under dynamic stimuli using a fresh porcine mandibular bone model. H) Photographs showing the Bio‐Oss granules remaining in the tooth extraction sockets after 24 h dynamic stimuli. I) Photographs showing the L‐DPZ2‐Bio‐Oss composite remaining in the tooth extraction sockets after 24 h dynamic stimuli.

Compared with the comminuted fractures of long bones, craniomaxillofacial bone defects pose greater challenges due to their complex geometries, ultimately leading to both mental and physical burdens on the patient.^[^ ^26^ ^]^ To reconstruct the ideal bony contour after trauma, maxillofacial bone augmentation surgery can be carried out using bone substitutes.^[^ ^27^ ^]^ The most commonly used bone defect regeneration scaffold materials are xenogenic bone substitutes such as Bio‐Oss.^[^ ^28^ ^]^ However, due to their poor cohesiveness, continuous hemorrhaging during surgery and postoperative physiological activities often leads to leakage and displacement of the bone substitutes at the bone defect site and ultimately impacts the outcome of the surgery. As a result, the combination of these bone substitutes with binder materials, such as pastes or gels,^[^ ^28^, ^29^ ^]^ was investigated; however, these reported materials were unable to fix the bone substitute firmly, and they were also unable to resist various external forces. Therefore, in light of the excellent adhesiveness of the L‐DPZ hydrogel, we mixed granules of the commonly used xenogenic bone substitute Bio‐Oss with our hydrogel (**Figure** **4**A), and evaluated the potential applicability of this composite in the spatial maintenance of bone substitutes in augmented bone regeneration.

As shown in Figure 4B, after mixing the L‐DPZ hydrogel with the Bio‐Oss granules, the hydrogel‐Bio‐Oss composite continued to exhibit a satisfactory injectability and viscoelasticity, allowing it to be reshaped into different forms by adjusting the hydrogel to Bio‐Oss ratio. These observations are of particular importance since they render the clinical operation more convenient, and indicate that the composite can be adapted to different conditions at the bone defect surface. Subsequently, Bio‐Oss granules were mixed with the L‐DP, L‐DPZ1, and L‐DPZ2 hydrogels to form equally sized rectangular blocks, and the mechanical properties of these blocks were investigated using uniaxial compression and three‐point bending tests (Figure 4C,D). As indicated by the obtained stress‐displacement curves, both the uniaxial compression strength and three‐point bending strength increased with the addition of ZIF‐8 (L‐DP‐Bio‐Oss < L‐DPZ1‐Bio‐Oss < L‐DPZ2‐Bio‐Oss). In addition, the significantly increased compressive strength of the L‐DPZ2‐Bio‐Oss composite indicated that the Bio‐Oss granules became stiffer after mixing with the L‐DPZ2 hydrogel, thereby suggesting the possibility of this composite exhibiting increased resistance to deformity during surgery. This phenomenon was attributed to the strong adhesiveness endowed by the interactions between L‐DOPA and ZIF‐8, which maximized the bridging between the L‐DPZ2 hydrogel and the Bio‐Oss granules, ultimately leading to greater stiffness and increased mechanical strength.^[^ ^28a^ ^]^

After demonstrating the mechanical properties of the L‐DPZ2‐Bio‐Oss composite, the ability of the hydrogel to protect the Bio‐Oss granules from blood and water flushing during surgeries was evaluated using in vivo and ex vivo pig jaw bone augmentation surgery models (Figure 4E,G). As shown in Figure 4A and Movie S5, Supporting Information, the L‐DPZ2‐Bio‐Oss composite exhibited good viscoelasticity and injectability. As shown in Figure 4F (first panel), implant exposure occurred after the insertion of the implant into the mandibular alveolar bone of live Bama micropigs due to the presence of insufficient horizontal alveolar bone at the implantation site. The injection of L‐DPZ2‐Bio‐Oss into the exposed implant and surrounding alveolar bone area was then carried out to reconstruct the bony contour (Figure 4F, second panel), after which, water flushing was carried out. Importantly, it was found that the hydrogel‐Bio‐Oss composite was anchored firmly in situ (Figure 4F, third panel and Movie S6, Supporting Information), thereby demonstrating the capacity of the L‐DPZ2 hydrogel to protect the bone granules from external disruption during surgery.

Alveolar ridge site preservation (ARSP) was subsequently carried out (Figure 4G) using a fresh pig mandible to evaluate the long‐term stability of the L‐DPZ2 hydrogel under dynamic stimuli (Movie S7, Supporting Information).^[^ ^30^ ^]^ ARSP is a bone augmentation surgery that involves filling the alveolar socket with biomaterials after tooth extraction to minimize alveolar bone resorption, thereby providing a sufficient bone mass and bone quality for implant placement at a later date.^[^ ^31^ ^]^ Thus, the second molars of porcine mandibles were extracted (Figure 4H,I, first panel) and the extraction sockets were filled with either Bio‐Oss granules (Figure 4H, second panel) or the L‐DPZ2‐Bio‐Oss composite (mixed with a blue dye) (Figure 4I, second panel), and covered with a Bio‐Gide collagen membrane. Dynamic food flow was then simulated in the mouth by stirring grain particles (sizes of 1.5–2 mm) in phosphate‐buffered saline, and the impact of the flowing particles against the Bio‐Oss and L‐DPZ2‐Bio‐Oss materials was used to simulate the shear force, friction, and impact occurring during mastication (Movie S7, Supporting Information). After 24 h of stirring, the Bio‐Gide specimen fell off, and very few Bio‐Oss granules could be observed in the extraction socket (Figure 4H, third panel). In sharp contrast, the majority of the L‐DPZ2‐Bio‐Oss composite remained in the socket after the Bio‐Gide cover was removed (Figure 4I, third panel). To further visualize the distribution of material remaining in the tooth extraction socket, cone‐beam computed tomography (CBCT) scanning was carried out. Indeed, the 3D CBCT images and CT values confirmed the macroscopic observations, wherein the Bio‐Oss granules detached completely from the socket, while the majority of the L‐DPZ2‐Bio‐Oss composite remained fixed to the alveolar bone in all dimensions after 24 h of continuous flushing (Figure 4H,I, fourth panel and Figure S2, Supporting Information). These results demonstrate that the L‐DPZ hydrogel shows potential for use as a binder that can facilitate the bone grafting of xenogenic bone substitutes whilst also protecting them from washing out by blood, water, and saliva.

### 2.4 Evaluation of the In Vitro and In Vivo Biocompatibility and Biodegradation Properties of the L‐DPZ Hydrogels

**Figure 5:**
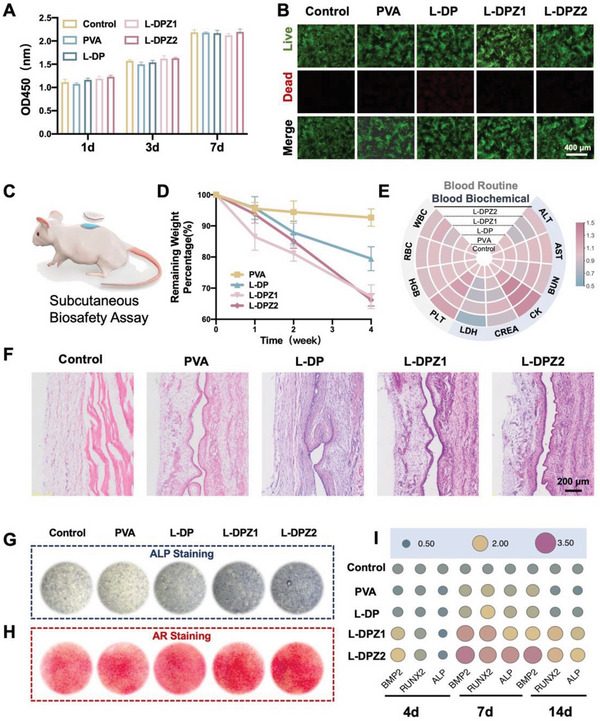
Evaluation of the in vivo and in vitro biocompatibility and biodegradation properties and the in vitro osteogenic ability of the hydrogels. A) Viability of MC3T3‐E1 cells treated with PVA, L‐DP, L‐DPZ1, L‐DPZ2, or the control for 1, 3, and 7 days (*n* = 3). B) Representative images of the Live/Dead staining of rBMSC cells after incubation with PVA, L‐DP, L‐DPZ1, L‐DPZ2, or the control for 7 days. C) Schematic illustration of the subcutaneous implantation model on the backs of SD rats. D) Weights of the PVA, L‐DP, L‐DPZ1, and L‐DPZ2 hydrogel tokens after implantation for 1, 2, and 4 weeks (*n* = 3). E) Heatmap of the levels of routine blood parameters (ALT, AST, BUN, CK, CREA, and LDH) and serum parameters (WBC, RBC, HGB, and PLT) in SD rats after treatment for 4 weeks (*n* = 3). F) H&E staining of the implantation sites after implantation with the PVA, L‐DP, L‐DPZ1, and L‐DPZ2 hydrogel tokens for 4 weeks. G) Representative ALP staining images of the rBMSCs after incubation with different hydrogels for 14 days. H) Representative AR staining images of the rBMSCs after incubation with different hydrogels for 21 days. I) Expression of the osteogenesis‐related genes, including BMP2, Runx2, and ALP, in the rBMSCs after incubation with different hydrogels for 4, 7, and 14 days (*n* = 3).

To investigate the in vitro biocompatibility of the L‐DPZ hydrogel, rat bone marrow stem cells (rBMSCs) were employed. More specifically, the proliferation of the rBMSCs was measured using a Cell Counting Kit‐8 (CCK‐8). As shown in **Figure** **5**A, compared with the control group, there was no significant difference in the viability of cells treated with the PVA, L‐DP, L‐DPZ1, and L‐DPZ2 hydrogels over 1, 3, and 7 days. Subsequently, a Live/Dead assay was carried out and a few dead cells (red) were detected in all groups after incubation for 7 days (Figure 5B). Taken together, these results indicate that the hydrogels exhibited no appreciable cytotoxicity.

To further investigate the in vivo biocompatibility and biodegradation behaviors of the L‐DPZ hydrogels, a subcutaneous implantation model was adopted using male Sprague Dawley (SD) rats (Figure 5C).^[^ ^1^, ^2^ ^]^ The rats were randomly divided into four groups (PVA, L‐DP, L‐DPZ1, and L‐DPZ2), and round tokens (1.3 cm diameter) of the relevant lyophilized hydrogels were implanted subcutaneously. After 1, 2, and 4 weeks, the implantation sites on the backs of the rats were reopened to measure the weights of the remaining hydrogel tokens. The appearances of the subcutaneously implanted hydrogels after 1, 2, and 4 weeks are presented in Figure S3, Supporting Information, wherein it can be seen that very little swelling and degradation occurred in the PVA‐treated group, while there was notable swelling and degradation in the L‐DP, L‐DPZ1, and L‐DPZ2 groups. Quantitatively, the PVA group exhibited a weight loss of only 7.33 ± 2.75% after 4 weeks of implantation. In contrast, the degradation rates of the L‐DP, L‐DPZ1, and L‐DPZ2 hydrogels after 4 weeks were 20.54 ± 3.86%, 32.75 ± 3.83%, and 33.77 ± 1.96%, respectively (Figure 5D). These results indicate the ability of the L‐DPZ hydrogels to withstand the in vivo physiological environment and to undergo stable degradation while assisting bone regeneration.

To measure the in vivo biocompatibility of the hydrogels, blood samples were collected for hematologic and biochemical evaluation after 4 weeks of subcutaneous implantation.^[^ ^9^ ^]^ As shown in Figure 5E, hydrogel implantation did not result in any significant changes in the levels of routine blood parameters, such as the white blood cell count (WBC), the red blood cell count (RBC), the hemoglobin (HGB) level, and the platelet level (PLT). In addition, the heart, liver, and kidney function indicators, including the serum levels of alanine transaminase (ALT), aspartate aminotransferase (AST), blood urea nitrogen (BUN), creatinine (CRE), creatine kinase (CK), and lactate dehydrogenase (LDH), also showed no significant alterations after different implantation times (Figure 5E). Furthermore, histological analysis was carried out for the tissues collected from around the implantation sites, wherein hematoxylin and eosin (H&E) staining showed no infiltration of inflammatory cells surrounding the implantation sites in any group (Figure 5F). Cumulatively, these findings demonstrate the excellent in vivo biocompatibility of the L‐DPZ hydrogel and its suitability for use in clinical applications.

### 2.5 In Vitro and In Vivo Osteogenic Activities of the L‐DPZ Bone Glues

To investigate the in vitro osteogenic activities of the L‐DPZ hydrogel, the rBMSCs were incubated with the different hydrogels, and after 14 days, the ALP activities and calcium mineral depositions were determined by alkaline phosphatase (ALP) and alizarin red (AR) staining, respectively.^[^ ^1^, ^32^ ^]^ As shown in Figure 5G, the degree of ALP staining, which represents an early marker of osteoblast metabolic activity,^[^ ^32^ ^]^ was significantly denser in the L‐DPZ1 and L‐DPZ2 groups than in the control, PVA, and L‐DP groups (Figure 5G). A similar trend was also observed for the AR staining (Figure 5H), wherein the cells treated with the L‐DPZ1 and L‐DPZ2 hydrogels exhibited a large number of red calcified nodules, while the distribution of the mineral nodules in the other three groups was relatively sparse.

In addition to the above macroscopic images of ALP and AR staining, the reverse transcription polymerase chain reaction (RT‐PCR) was conducted to quantitatively assess the osteogenic activity stimulated by the L‐DPZ hydrogels at the gene expression level. For this purpose, the expression levels of three representative osteogenesis‐related genes, namely BMP‐2, Runx2, and ALP, were investigated after culturing the rBMSCs with the hydrogels for 4, 7, and 14 days.^[^ ^1b^ ^]^ As depicted in Figure 5I, at day 4, the expression levels of BMP‐2 and Runx2 were highest in the L‐DPZ2 group, followed by the L‐DPZ1 group. Additionally, on days 7 and 14, the cells treated with the L‐DPZ2 hydrogel exhibited the highest expression level of all tested genes (BMP‐2, Runx2, and ALP) compared with the other four groups. As expected, these results confirmed that the addition of ZIF‐8 increased the in vitro osteogenesis of the L‐DPZ hydrogel, and the effect was amplified upon increasing the concentration of ZIF‐8, demonstrating that the Zn^2+^ released from ZIF‐8 provided a favorable microenvironment for osteogenesis.^[^ ^1^, ^13^ ^]^

**Figure 6:**
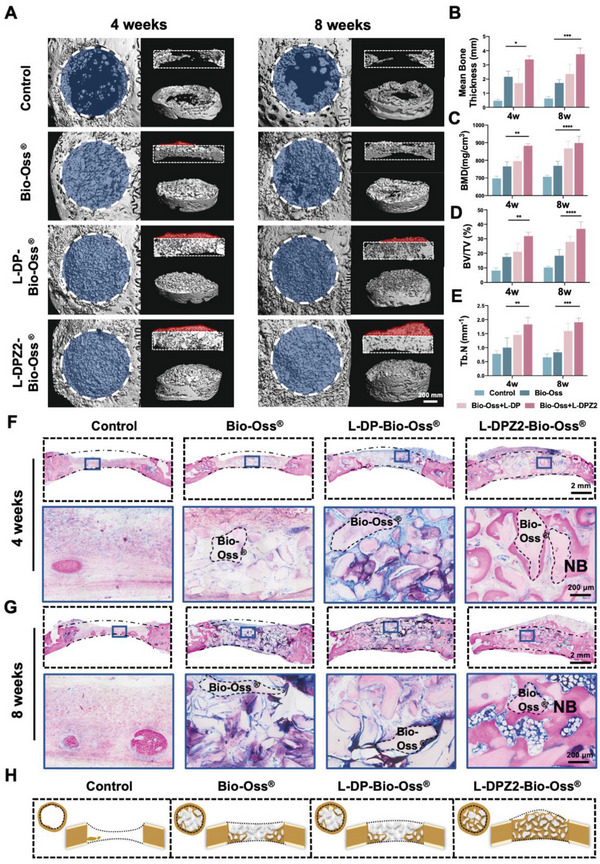
In vivo evaluation of bone regeneration after L‐DPZ2‐Bio‐Oss treatment of rabbit calvarial bone defects. A) Representative 3D micro‐CT images of the rabbit calvarial bone defects after 4 and 8 weeks of treatment with the different hydrogels. Variation in the B) bone thickness, C) BMD, D) BV/TV, and E) Tb.N. parameters after 4 and 8 weeks of treatment with the different hydrogels (*n* = 3). F,G) VG staining of the rabbit calvarial bone defects after treatment with the different hydrogels for F) 4 and G) 8 weeks. H) Schematic diagram of new bone formation in the sagittal and transverse sections of the control, Bio‐Oss, L‐DP‐Bio‐Oss, and L‐DPZ2‐Bio‐Oss groups (brown: bone tissue, gray: Bio‐Oss, dotted line: bone plate outline). *p < 0.05, **p < 0.01, ***p < 0.001, ****p < 0.0001, ns, not significant. Data were analyzed using one‐way ANOVA with Tukey's post hoc analysis.

As described previously with reference to Figure 4, the L‐DPZ hydrogels were mixed with Bio‐Oss to form a homogeneous mixture with improved strength, and the resistance of this composite to external forces was tested in the maxillofacial bone augmentation surgery and in the ARSP surgery of pigs. Although Bio‐Oss only possesses a bone conduction effect, it was considered that the osteogenic induction ability of the L‐DPZ hydrogels in vitro could compensate for this deficiency. Thus, to further examine the osteogenic capability of the L‐DPZ hydrogel and its capacity to aid Bio‐Oss retention in vivo, a rabbit calvarial critical‐size defect model was established^[^ ^33^ ^]^ and bone augmentation surgery was conducted. Considering the superior performance of the L‐DPZ2 hydrogel compared with the L‐DPZ1 hydrogel in the above experiments, the L‐DPZ2 hydrogel was used for investigation in the in vivo model. The calvarial defects were filled with either Bio‐Oss, the L‐DP‐Bio‐Oss composite, the L‐DPZ2‐Bio‐Oss composite, or no material (control) (Figure S4A,B, Supporting Information). The rabbits were euthanized 4 and 8 weeks following the procedure, and the hearts, livers, spleens, lungs, and kidneys were collected at 8 weeks for HE staining analysis. Indeed, as shown in Figure S4D, Supporting Information, all treatment groups exhibited good biocompatibility. Subsequently, micro‐computed tomography (micro‐CT) was used to evaluate the retention of bone granules and the effect of bone augmentation in each group at times of 4 and 8 weeks following the procedure (**Figure** **6**A). The vertical view of the reconstructed micro‐CT images (Figure 6A, left panels) revealed that the Bio‐Oss group experienced some granule loss, which was likely caused by blood flow‐induced erosion and a lack of tight binding of the Bio‐Oss granules. In contrast, the L‐DP‐Bio‐Oss and L‐DPZ2‐Bio‐Oss composite groups displayed denser filling properties and minimal loss of the treatment granules. The most visible effect of bone augmentation (highlighted in red) was observed for the L‐DPZ2‐Bio‐Oss experiment after 4 and 8 weeks, as shown in the sagittal plane and the 45° top view of the repaired defect area (Figure 6A, right panels). However, neither the Bio‐Oss group nor the L‐DP‐Bio‐Oss group exhibited sufficient bone thickness, so the effects of the hydrogels were further investigated by means of quantitative analysis. As presented in Figure 6B, after 4 weeks, the mean bone thickness of the L‐DPZ2‐Bio‐Oss group was 7.2, 1.6, and 1.9‐fold higher than in the control (0.47 ± 0.11 mm), Bio‐Oss (2.16 ± 0.40 mm), and L‐DP‐Bio‐Oss (1.71 ± 0.98 mm) groups (Figure 6B). In addition, after 8 weeks, the thickness was also markedly higher after treatment with the L‐DPZ2‐Bio‐Oss composite than with the other treatments (Figure 6B). Furthermore, bone mineral density (BMD), bone volume/total volume ratio (BV/TV), and trabecular number (Tb. N) are also important parameters for evaluating the quality of bone tissue.^[^ ^32^, ^34^ ^]^ As shown in Figure 6C–E, superior bone analysis parameters were observed for the L‐DPZ2‐Bio‐Oss composite group compared to the other three groups, after both 4 and 8 weeks of implantation.

Van Gieson's (VG) picrofuchsin staining was then used to histologically reveal the quality of newly formed bone, whereby osteoid was stained blue and mature bone tissue was stained red.^[^ ^33^, ^35^ ^]^ After 4 and 8 weeks, clear fiber encapsulation and very limited new bone growth could be seen in the control group (Figure 6F). Despite the presence of Bio‐Oss granules in the Bio‐Oss and L‐DP‐Bio‐Oss groups, the granules were mostly surrounded by fibers rather than newly formed bone, with a new bone area of <3% being recorded for both groups. However, upon the incorporation of the L‐DPZ2 hydrogel, the bone‐inducing function of the ZIF‐8 nanoparticles led to the creation of newly woven bone tissue around the majority of Bio‐Oss granules at 4 weeks, which corresponded to a new bone formation area of 17.66 ± 2.72%; after 8 weeks, this reached 41.06 ± 1.84% (Figure 6F,G and Figure S4C, Supporting Information). Compared to the other groups, the L‐DPZ2‐Bio‐Oss group not only exhibited superior horizontal new bone ingrowth but also demonstrated more stable space maintenance of the bone plate thickness and morphology, as illustrated in Figure 6H.

As expected, these experiments indicate that the bone defects treated with the combination of Bio‐Oss granules and the L‐DPZ2 hydrogel exhibited the best osteogenesis outcome. Based on these results, several potential mechanisms were proposed for the superior osteogenic performance of the L‐DPZ hydrogel, including the excellent adhesiveness endowed by the catechol groups of L‐DOPA,^[^ ^36^ ^]^ which, together with the catechol–metal coordination bonds present between L‐DOPA and ZIF‐8,^[^ ^37^ ^]^ ensured strong fixation of the Bio‐Oss granules in the bone defect area. This phenomenon led to long‐term in situ stability for the bone granules, thereby preventing their removal by the flow of blood or body fluids during the surgical procedure.^[^ ^38^ ^]^ In addition, the appropriate degradation rate of the L‐DPZ hydrogel enabled sufficient bone tissue regeneration to bridge the bone defects. According to a previous study, Zn^2+^ ions are released both intracellularly and extracellularly by the ZIF‐8 nanoparticles, so it is also possible that endocytosis receptors for these species may play a role in inducing the canonical mitogen‐activated protein kinase pathway to promote osteogenesis in the rBMSCs.^[^ ^39^ ^]^

### 2.6 Different Patterns of New Bone Formation in the Calvarial Defect Group Treated by the L‐DPZ2‐Bio‐Oss Composite

**Figure 7:**
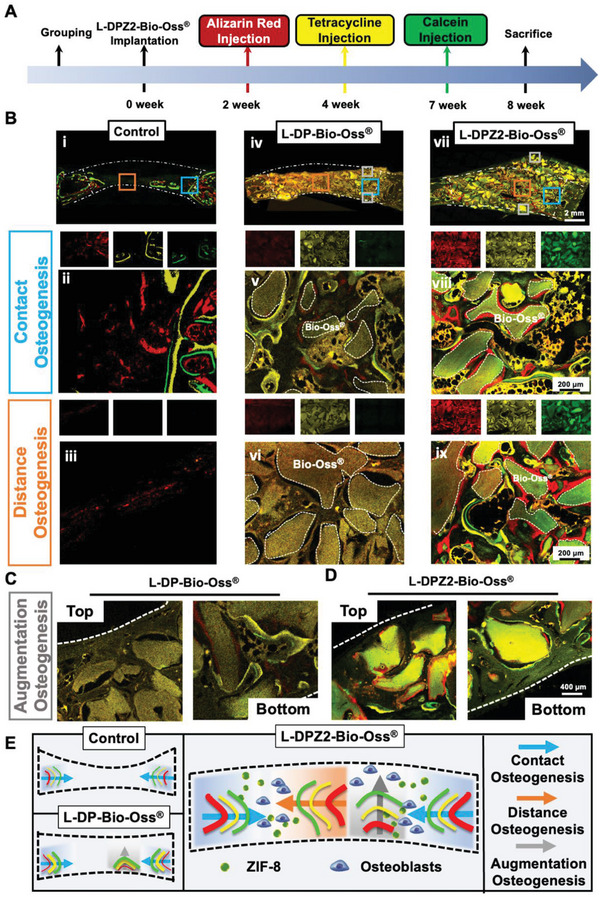
Evaluation of the spatiotemporal behavior of the newly formed bone after L‐DP‐Bio‐Oss treatment. A) Schematic showing the timeframe for intramuscular injection of mineral dyes in in vivo bone formation studies. B) Sequential fluorescent labeling observation showing new mineral deposition in rabbit calvarial bone defects treated with L‐DP‐Bio‐Oss (iv–vi) and L‐DPZ2‐Bio‐Oss (vii–ix), or no filler (control) (i–iii) (red: Alizarin red, week 2; yellow: Tetracycline hydrochloride, week 4; green: Calcein, week 7; dotted line: threads of Bio‐Oss). High‐magnification fluorescently labeled images of the bottom and top portions of defects treated with C) L‐DP‐Bio‐Oss or D) L‐DPZ2‐Bio‐Oss. E) Schematic diagrams showing the osteogenesis modes (contact, distance, and augmentation osteogenesis) for defects treated with L‐DP‐Bio‐Oss, L‐DPZ2‐Bio‐Oss, or no hydrogel (control).

To gain additional insight into the spatiotemporal behavior of the newly formed bone, a sequential fluorescence labeling assay was carried out.^[^ ^35^, ^40^ ^]^ More specifically, intramuscular injections of fluorescently labeled mineral dyes were administered at predetermined periods (**Figure** **7**A). As shown in Figure 7Bii–vi), the control and L‐DP‐Bio‐Oss groups exhibited only weak and dispersed red fluorescence, indicating that almost no new bone was growing inward at 2 weeks, thereby indicating that the osteogenesis process was delayed. In contrast, when the L‐DPZ2‐Bio‐Oss material was employed, a more extensive and intensive red fluorescence was observed surrounding the Bio‐Oss, suggesting that bone mineral deposition occurred just 2 weeks after surgery (Figure 7Bviii,ix). In addition, as shown in Figure 7Bi–vi), in the control and L‐DP‐Bio‐Oss groups, yellow and green fluorescent regions (late period new bone) were present at both ends of the defect (contact osteogenesis), while little to no new bone was seen to grow from the center of the defect to both ends (distance osteogenesis). Amazingly, L‐DPZ2 supplementation led to the bidirectional bone formation by enabling both contact osteogenesis and distance osteogenesis (Figure 7Bviii,ix). Intriguingly, application of the Bio‐Gide collagen membrane to the defect region after surgery prevented osteogenesis of the periosteum,^[^ ^41^ ^]^ resulting in the observation of new bone only at the base of the defect in the L‐DP‐Bio‐Oss group (Figure 7C); according to previous studies, this may be attributed to osteogenesis of the dura.^[^ ^42^ ^]^ In contrast, the L‐DPZ2‐Bio‐Oss group demonstrated full‐thickness osteogenesis from bottom to top, despite the absence of a periosteum, ultimately resulting in a totally internalized Bio‐Oss bone augmentation effect (Figure 7D). Overall, the L‐DPZ2‐Bio‐Oss group outperformed the other two groups in the three crucial aspects, exhibiting an enhanced early osteogenesis capacity (red fluorescence area), the coexistence of contact and distance osteogenesis (blue and orange arrows), and augmented osteogenesis from bottom to top (gray arrows), as illustrated in Figure 7E.

Upon examination of the three new bone formation patterns shown in Figure 7, it was clear that the L‐DPZ2‐Bio‐Oss group demonstrated the optimal osteointegration effect. This was initially attributed to the fact that L‐DOPA, the adhesive component of the L‐DPZ hydrogel, actively facilitated the migration and adherence of the rBMSCs to the defect area,^[^ ^43^ ^]^ which created favorable conditions for early osteogenesis (i.e., 2–4 weeks) when combined with the osteogenic induction effect of the ZIF‐8 nanoparticles. Moreover, the homogeneous mixing of L‐DPZ2 and Bio‐Oss allowed the ZIF‐8 nanoparticles to tightly encase Bio‐Oss at both the edge and center sites of the defect, which not only ensured a stable bone deposition environment but also encouraged active bone regeneration in the center and top areas, where osteogenesis is traditionally difficult.

## 3 Conclusion

In this study, an ultra‐high‐strength orthopedic adhesive (L‐DPZ, L‐DOPA‐PVA‐ZIF‐8) was developed for use as a multi‐functional tool to splice bone fragments and adhere bone grafts, whilst also guiding and promoting subsequent bone healing during the matrix degradation process. More specifically, this adhesive integrates PVA, L‐DOPA, and ZIF‐8. As a result, the combination of noncovalent and covalent bonds, along with metal–polyphenol chelation, the novel adhesive was able to adapt to complex clinical scenarios via excellent injectability, self‐healing properties, and ultra‐high mechanical strength. Furthermore, the prepared L‐DPZ hydrogel exhibited extremely strong adhesion properties with bovine bone samples, luxated teeth, and teeth fragments in vitro, and it was also found to form a homogeneous mixture with Bio‐Oss, a typical bone graft material. Moreover, the L‐DPZ hydrogel showed good biocompatibility and an appropriate degradation rate. Importantly, the anchoring effect of an L‐DPZ‐Bio‐Oss composite was demonstrated using a rabbit calvarial bone augmentation model, which also confirmed the inducing effect of L‐DPZ on bone matrix deposition, as evidenced by an increased bone thickness and new bone ingrowth at the defect site. These results, therefore, indicate that the prepared L‐DPZ superglue provides a novel solution for various clinical dilemmas such as the fixation of highly comminuted bone fractures, the reconstruction of complicated bone defects, and the replantation of challenging teeth dislocations.

## 4 Experimental Section

### Materials

PVA (*M*_w_ 85–124 kDa) and 3,4‐dihydroxy‐DL‐phenylalanine (L‐DOPA) were purchased from Sigma Aldrich (USA). ZIF‐8 NPs were bought from Feynman Nanomaterials Technology Co., LTD (China). Resorbable bone materials (Bio‐Oss) and resorbable collagen membranes (Bio‐Gide) were purchased from Geistlich (Switzerland). MEM Alpha Modification (*α*‐MEM), DMEM, penicillin‐streptomycin, phosphate‐buffered saline (PBS), trypsin, and fetal bovine serum (FBS) were purchased from Gibco BRL (Gaithersburg, USA).

### Synthesis of the Hydrogels

This was carried out according to the previous study.^[^ ^1^ ^]^ For the preparation of the L‐DP hydrogel, PVA (48 mmol) was dissolved in DMSO (120 mL) at 100 °C, and 6 g of NaHSO_4_·H_2_O was then added to the PVA solution. After decreasing the temperature to 80 °C, L‐DOPA (8 mmol) was added and the reaction was kept at 80 °C for 24 h under N_2_. After that, the solution was purified by dialysis for 3 days using a dialysis membrane (MWCO 3500 Da, Biosharp, China). The final product was freeze‐dried and stored under vacuum. For the preparation of L‐DPZ hydrogels, the L‐DP hydrogel was first dissolved in ultra‐purified water at a concentration of 200 mg mL^−1^. Then, the ZIF‐8 powder was dissolved in ultra‐purified water to achieve the mass ratios (L‐DP/ZIF‐8) of 100/1 or 100/2. Immediately, equal volumes of the two solutions were homogenously mixed to get the final L‐DPZ hydrogels. Hereafter, the hydrogels modified by ZIF‐8 NPs were designated L‐DPZ1 and L‐DPZ2 according to different mass ratios 100/1 and 100/2, respectively.

### Characterization of the Hydrogels

The morphology and EDS mapping of the prepared hydrogels were performed using SEM (FEI Hillsboro, USA). The synthesis of the L‐DP, L‐DPZ1, and L‐DPZ2 polymers was confirmed by means of FTIR (Thermo Nicolet, USA). The elemental compositions of each group of hydrogels were characterized by XPS (ESCALAB250Xi, Thermo Scientific, USA).

### Rheological Measurements and Self‐Healing Performance

The rheological properties of hydrogels were assessed using a rheometer (Anton Paar, Austria). All the experiments were carried out at 25 °C using a 40 mm parallel plate with a plate gap of 1 mm. Frequency sweeps were conducted at oscillation frequencies from 0.1–1000 rad s^−1^ under a 1% strain level. Strain sweeps had a fixed oscillation frequency of 10 rad s^−1^ and variable applied strain of 0.1–1000%. To observe the damage‐healing properties of the hydrogels, the storage modulus (*G*′) and loss modulus (*G*″) of the hydrogel were measured by a continuous step change of the strain between high strain 600% and 1% with a frequency of 10 rad s^−1^. An additional demonstration of self‐healing capacity was carried out by cutting two hydrogel disks dyed with different colors in half and reconnecting them after exchanging red and blue halves. Tensile stress was manually applied with tweezers to the reconnected disks after allowing healing for 30 min.^[^ ^2^ ^]^

### Mechanical Tests

Adhesion strength was determined on bovine cortical bone specimens, which were cuboids of 50 × 10 × 10 mm or 80 × 10 × 2 mm cut from bovine femurs. Before testing, 50 µL of hydrogel was injected into the bone pieces and the other piece was connected in both end‐to‐end and lap shear manners. After 2 h of solidification, tensile adhesion strength tests were carried out using a universal material testing machine (INSTRON, UA) in both end‐to‐end and lap shear directions to record the maximum stress of bonded joints before failure. The adhesive properties of hydrogels to PTFE or glass sheets were tested with the same lap shear method.

### Ex Vivo Bovine Fracture Tooth Model

To assess the ability of bone glues to bind fractured teeth, experiments were performed using fresh bovine jaws purchased from a local butcher. A saw blade grinds the upper third of an incisor tooth to simulate a broken tooth. The L‐DPZ2 bone glue was injected into the cross‐section. Then the two ends were placed together. After 24 h, the fractured end of the tooth was perforated with a drill to suspend the weight.

### Ex Vivo Porcine Dislocated Tooth Bonding Reduction Model

To evaluate the effect of bone glues on the adhesive restoration of lost teeth, a fresh pig jaw purchased from a local butcher was used in the experiment. The mandibular first tooth of the pig was extracted, the tooth socket was scratched, L‐DPZ2 bone glue was injected into the tooth socket, and the extracted tooth was inserted back into the extraction site again. After 24 h, the crown of the tooth was perforated with a drill to suspend the weight.

### In Vivo Rabbit Femoral Comminuted Fracture Model

To evaluate the fracture reduction ability of the L‐DPZ2 bone glue for comminuted fractures, a New Zealand rabbit was used.^[^ ^3^ ^]^ After anesthesia, a skin incision was made in the tibial tuberosity region at the metaphysis or diaphysis of the tibia and extended to the underlying fascia and periosteum. A 2 mm single cortical hole was drilled intermittently in the cortex and cancellous bone below the patellar ligament, and a 2 mm × 15 mm Kirschner wire was inserted into the marrow cavity. After the femur was crushed slightly above the metaphysis of the femoral diaphysis with a rongeur, each part was reduced by bonding with L‐DPZ2 bone glue at the broken end of the fracture.

### In Vivo Porcine Implant Exposure Model

To assess the wet adhesive ability of the L‐DPZ hydrogel after mixing with bone granules, Bama miniature pigs were used in the following experiments. Under general anesthesia, the gingival tissue in the space between the canine and premolars teeth of a Bama miniature pig was cut open, and the thin alveolar bone was exposed. A 3.3 × 8 mm implant (Trausim, China) was implanted, and the implant was exposed by 3 mm. After injecting a mixture of L‐DPZ2 bone glue and Bio‐Oss onto the exposed area of the implant, saline was sprayed into the area using a syringe.

### Mechanical Test of the Hydrogel and Bio‐Oss Composite

To evaluate the mechanical properties of the hydrogel and Bio‐Oss composite, three‐point bending and compression tests were performed. First, the Bio‐Oss and hydrogel were mixed at the ratio of 100mg:200 uL, and then the mixture was made into a 5 mm × 5 mm × 12 mm cuboid with a mold. After solidification for 2 h, the cuboid was tested with a universal mechanical tester. The compression test was performed at a loading rate of 1 mm min^−1^ to record the maximum load achieved by the specimen at fracture. In the three‐point bending experiment, the bending span was 4 mm, the bending speed was 1 mm min^−1^, and the maximum load before failure was recorded.

### Ex Vivo Porcine Site Preservation Model

To evaluate the ability of bone glues mixed with Bio‐Oss to preserve extraction socket sites, experiments were performed using fresh pig jaws purchased from a local butcher. Bilateral mandibular second premolars were extracted. A socket on one side of the jaw was filled with Bio‐Oss mixed with normal saline, and a socket on the other side was filled with the same amount of L‐DPZ2‐Bio‐Oss composite and covered with a medical collagen sponge (Kejibang, China). Then, the mandible was placed in a beaker filled with PBS, and quinoa particles were added under constant stirring to simulate the oral feeding environment.^[^ ^4^ ^]^

### Cell Culture Study

rBMSCs were obtained from the long bone marrow of 3‐week‐old SD rats. The rBMSCs were cultured in 10 cm culture dishes (Corning, USA) containing *α*‐MEM supplemented with 10% FBS and 1% PS in a humidified atmosphere of 5% CO_2_ at 37 °C. Passage 3 rBMSCs were used in experiments. To evaluate the biocompatibility of the hydrogel, rBMSCs were seeded in the wells of a 24‐well microtiter plate at a cell density of 2 × 10^4^ cells per mL, and 50 µL of PVA, L‐DP, L‐DPZ1 or L‐DPZ2 hydrogels at a concentration of 50 mg mL^−1^ were added to each well. The control group refers to wells without adding any hydrogel. The cell proliferation was measured with the CCK‐8 (Solarbio, China) assay, and the results were acquired after 1, 3, and 7 days.

### Subcutaneous Implantation Model

SD rats (male, 6–8 weeks old, 180–220 g) were used for the subcutaneous implantation of different hydrogels and for an in vivo injectability test. For the implantation test, after anesthesia using an inhalation anesthesia apparatus (Litian, China), the skin on the rats’ back was shaved and disinfected and a transverse incision was made on the neck. Subcutaneous pockets were created with blunt forceps and disks of the PVA, L‐DP, L‐DPZ1, and L‐DPZ2 hydrogels with a diameter of 13 mm and a thickness of 3 mm were implanted in the pocket. Intramuscular injection of penicillin was applied for 3 days after surgery.

At certain time points after surgery, rats were euthanized and the subcutaneous implant site was dissected and exposed for gross observation. For pathological evaluation, the hydrogel disks along with the surrounding tissue were collected and fixed for staining. After fixation, samples were sliced in thickness of 8 µm and further subjected to H&E. For the characterization of degradation, the hydrogel disks were carefully dissected to remove the surrounding tissue, and freeze‐dried for weighing. The remaining weight percent at different time points was calculated using the following formula based on the remaining dry weight (*W*_1_) and the initial dry weight of the hydrogel disks (*W*_0_).^[^ ^3^, ^5^ ^]^

(1) $$\mathrm{Remaining}\;\mathrm{weight}\;\mathrm{percentage}=\left(W_{0}-W_{1}\right)/W_{0}\times 100\%$$

Meanwhile, blood and serum samples of rats were collected for 4 weeks, and the results were normalized by the control group.

### In Vitro Osteogenesis‐Related Research

The osteogenic differentiation *α*‐MEM medium contained *β*‐glycerophosphate (10 × 10 ^−3^ m), L‐ascorbic acid (50 µg mL^−1^), and dexamethasone (10 × 10^−9^ m) (osteogenic medium). First, rBMSCs were seeded in 24‐well plates at a density of 2 × 10^4^ cells per mL and incubated overnight, and then the differentiation medium was added. The medium was refreshed every 2 days. For investigating the extent of osteogenic differentiation, rBMSCs cells were cultured in an osteogenic medium for 14 days. To measure the ALP expression, cells were fixed with 4% PFA and stained using the BCIP/NBT ALP color development kit (Roche, Switzerland). The mineralization of rBMSCs cells was assessed on day 21 using alizarin red staining. The cells were fixed with 4% PFA and stained with 1% alizarin red solution (Sigma) for 30 min. An optical microscope was used to observe the ALP and AR staining. The expression of osteogenic‐related genes in rBMSCs was analyzed by RT‐PCR. The rBMSCs were cultured in differentiation media at a cell density of 2 × 10^4^ cells per mL for 4, 7, or 14 days. After this, the cells were washed twice with PBS, and the total RNA was extracted and reverse‐transcribed into complementary DNA. ALP, BMP2, and RUNX2 mRNA levels were quantified by using SYBR Premix Ex TaqII. The RT‐PCR primer sequences are listed in Table S1, Supporting Information. These relative expression level genes were normalized relative to *β*‐actin.^[^ ^6^ ^]^ For statistical analysis, the control group was used for normalization.

### Critical‐Sized Rabbit Calvarial Bone Defect Model

New Zealand rabbits (female, 10–12 weeks old, 2.5‐3 kg) were used for the experiment. To perform a critical‐sized cranial defect model, the rabbits were anesthetized using an inhalation anesthesia apparatus (Litian, China), and the skin on the cranial region was shaved and disinfected. Then, a sagittal incision was made on each rabbit's scalp and the calvarium was exposed by blunt dissection.^[^ ^7^ ^]^ Two bilateral defects were created per rabbit by using a 10 mm diameter trephine bur. Then 100 mg of Bio‐Oss and 200 µL of the L‐DP or L‐DPZ2 hydrogels were mixed and loaded into the defects for the L‐DP and L‐DPZ2 groups, and 100 mg of Bio‐Oss was mixed with 200 µL of normal saline and loaded into the defects for the Bio‐Oss group. The control group had no material loaded into the defects. The defects were then covered with Bio‐Gide collagen membranes (Geistlich, Switzerland) and carefully sutured. 4 or 8 weeks after the surgery, the animals were sacrificed.

### Microcomputed Tomography Analysis

Calvarial bone defect samples were fixed in 4% paraformaldehyde overnight and then analyzed by a Micro CT (SANCO Medical AG, Switzerland). The scanning condition was 70 kV and 112 µA with a thickness of 0.048 mm per slice in medium‐resolution mode 1024 reconstruction matrix and 200 ms integration time. The threshold values of bone were set as 315 to 543 Hounsfield units. To determine the volume of interest, a 10 mm diameter circular contour was drawn around the center of each defect. The BV/TV ratio, BMD, and Tb. N were analyzed.^[^ ^8^ ^]^

### Hard Tissue Sections Preparation and Histology Staining

After radiological examination, the specimens were embedded in methyl methacrylate, and the hard tissue sawing system (E200CP, EXAKT Verteriebs, Germany) was used to prepare 20‐um thick sections. According to the previous studies,^[^ ^9^ ^]^ the sections were stained with VG staining.

### Sequential Fluorescent Labeling

According to the previous studies,^[^ ^9^ ^]^ a polychrome sequential fluorescent labeling method was used to elucidate the process of new bone formation and mineralization. At 2, 4, and 7 weeks postoperatively, different fluorescein‐labeled composites were administered intramuscularly in a sequence of 30 mg kg^−1^ Alizarin Red S, 30 mg kg^−1^ tetracycline hydrochloride, and 30 mg kg^−1^ calcein, respectively.

### Statistical Analysis

Statistical evaluation was performed using one‐way ANOVA with Tukey's post hoc analysis using GraphPad Prism software (version 9.0.0). The data of RT‐PCR, blood routine, and blood biochemical indexes were normalized with the control group as the standard. Sample size (*n*) for each statistical analysis = 3. Data were expressed as means ± SD. *p* values less than 0.05 were considered significantly significant. *p < 0.05, **p < 0.01, ***p < 0.001, ****p < 0.0001; ns, not significant.

### Ethical Statement

All animal experiments were performed according to protocols approved by the Chongqing Medical University Ethics Review Committee (CQHS‐REC‐2022‐108).

## Conflict of Interest

The authors declare no conflict of interest.

## Supporting information

Supporting Information

Supplemental Movie 1

Supplemental Movie 2

Supplemental Movie 3

Supplemental Movie 4

Supplemental Movie 5

Supplemental Movie 6

Supplemental Movie 7

## Data Availability

The data that support the findings of this study are available from the corresponding author upon reasonable request.
