# Supplementary material for: A Mechanically Reinforced Super Bone Glue Makes a Leap in Hard Tissue Strong Adhesion and Augmented Bone Regeneration
Source: Adv Sci (Weinh). 2023 Jan 25;10(11):2206450. doi: 10.1002/advs.202206450 (PMC10104643; doi:10.1002/advs.202206450)
Supplement: Supplementary file 1 — Supporting Information [file ADVS-10-2206450-s002.pdf]

ADVANCED SCIENCE
Open Access

Supporting Information

for *Adv. Sci.*, DOI 10.1002/advs.202206450

A Mechanically Reinforced Super Bone Glue Makes a Leap in Hard Tissue Strong Adhesion and Augmented Bone Regeneration

Shanshan Hu, Shan Wang, Qingqing He, Dize Li, Liangjing Xin, Chuanhang Xu, Xingyu Zhu, Li Mei, Richard D. Cannon, Ping Ji, Han Tang* and Tao Chen**

Supporting Information

A Mechanically Reinforced Super Bone Glue Makes a Leap in Hard Tissue Strong Adhesion and Augmented Bone Regeneration

Shanshan Hu#, Shan Wang#, Qingqing He, Dize Li, Liangjing Xin, Chuanhang Xu, Xingyu Zhu, Li Mei, Richard D. Cannon, Ping Ji, Han Tang*, and Tao Chen**

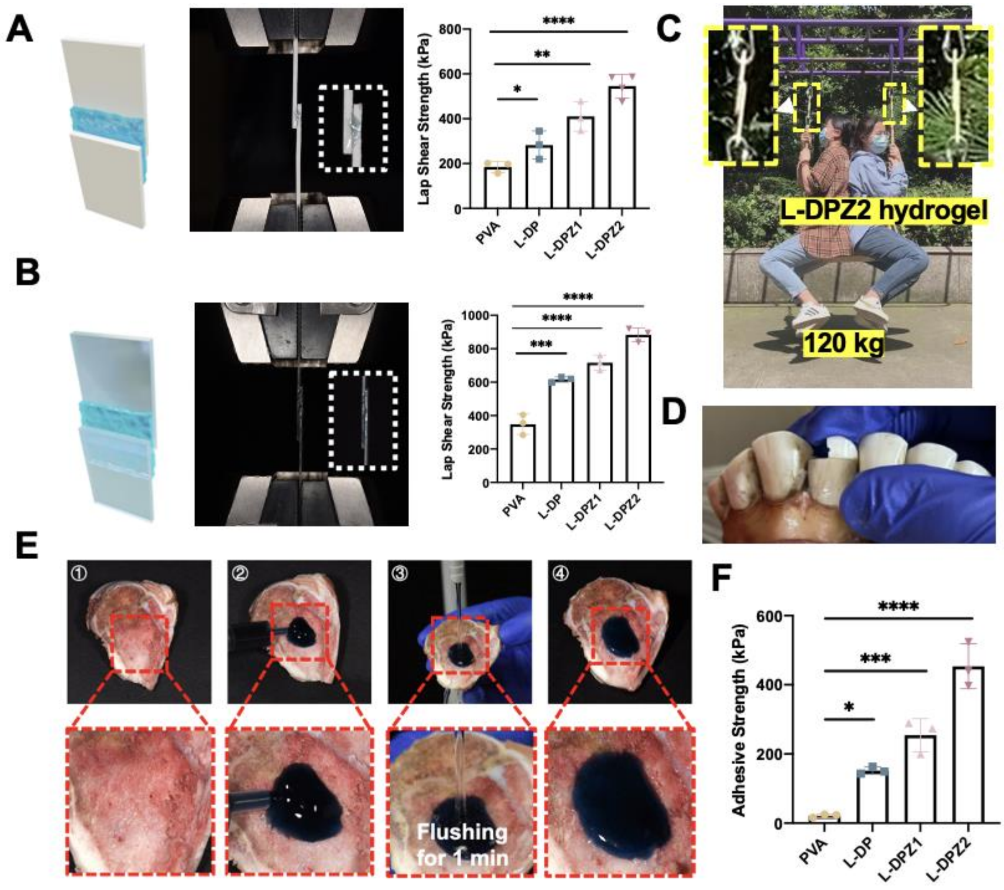

Figure S1. (A) Lap shear adhesion strength of different hydrogels on PTFE. (B) Lap shear adhesion strength of different hydrogels on glass. (C) Lift-up of two adult women weighing approximately 120 kg using two bonded bovine bone plates in area of 4 cm × 3 cm with L-DPZ2 hydrogel. (D) Gross view of tooth fracture but not adhesive surface failure after suspension of dumbbells. (E)Adhesion of the L-DPZ2 hydrogel (mixed with a blue dye) to the head of a pig femur under running water for 1 min. (F) Instant adhesive strength of different hydrogels on wet bone tissue. *P < 0.05, **P < 0.01, ***P < 0.001, ****P < 0.0001, ns, not significant. Data analyzed using one-way ANOVA with post hoc analysis.

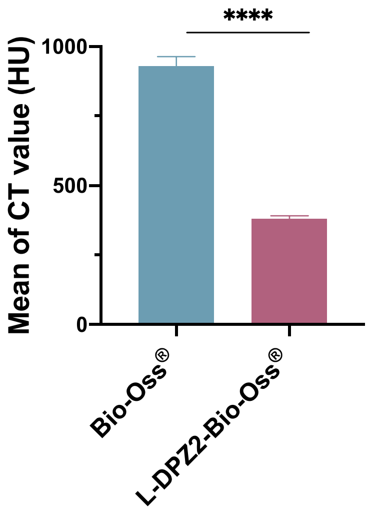

Figure S2. CT values of Cone Beam Computed Tomography (CBCT) of the tooth extraction sockets. *P < 0.05, **P < 0.01, ***P < 0.001, ****P < 0.0001, ns = not significant. Data were analyzed using t tests.

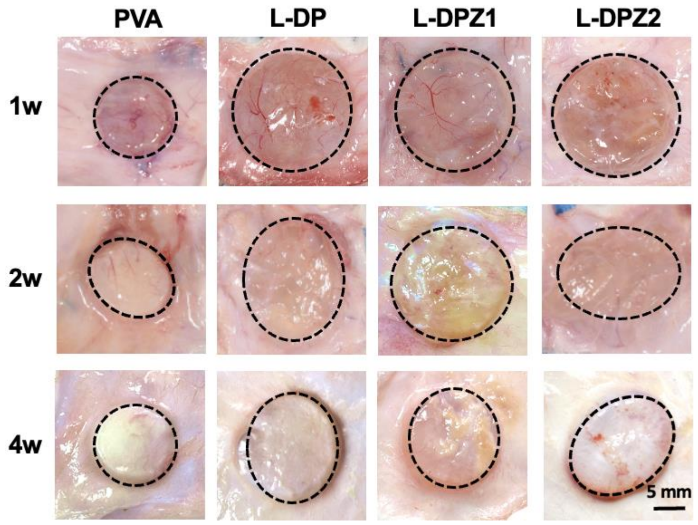

Figure S3. Gross view of implanted samples at subcutaneous site at 1 week, 2 weeks, 4 weeks.

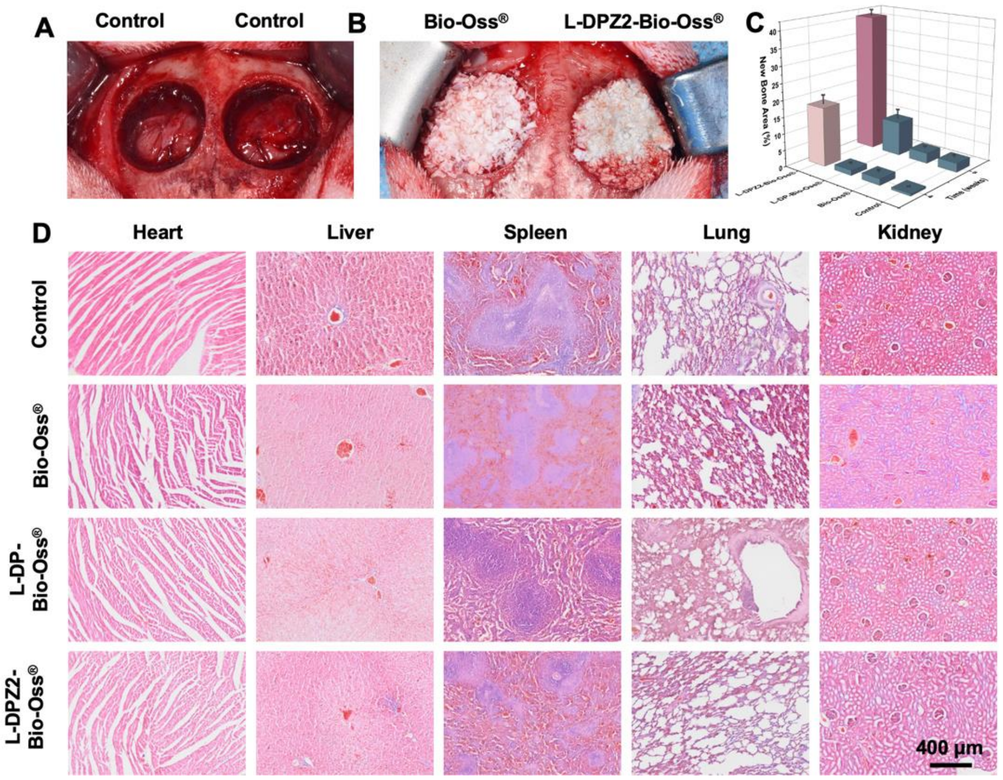

Figure S4. (A, B) Surgical gross view of calvarial defect in rabbits. (C) The semiquantitative analysis of new bone formation based on VG staining. (D) Histological images of heart, liver, spleen, lung, and kidney collected from different groups at 8 weeks postoperatively.

Gene (Rat)	Forward Primer (5′ to 3′)	Reverse Primer (5′ to 3′)
BMP2	ATGTGAGGATTAGCAGGTCTTTG	CTCGTTTGTGGAGTGGATGTC
RUNX2	GGCGTATTTCAGATGATGACACT	AAGTGAAGGTGGCTGGATAGTG
ALP	AGATGTGGCGGTCTTTGC	TCAGAACAGGGTGCGTAGG
β -actin	ACGGTCAGGTCATCACTATCG	GGCATAGAGGTCTTTACGGATG

Table S1. Primers for Quantitative real-time PCR.

Movie S1. Weight bearing after bonding of bovine bone slices with L-DPZ2.

Movie S2. Weight bearing after adhesion of luxated tooth with L-DPZ2.

Movie S3. Adhesion and anti-water flushing ability of L-DPZ2 in wet environment.

Movie S4. Instant wet bone tissue adhesion of L-DPZ2.

Movie S5. Injectablity of L-DPZ2-Bio-Oss®.

Movie S6. Bone augmentation surgery for peri-implant bone defect using L-DPZ2-Bio-Oss®.

Movie S7. Alveolar Ridge Site Preservation surgery after teeth extraction using Bio-Oss® or L-DPZ2-Bio-Oss®.

Reference:

[1] S. Hu, X. Pei, L. Duan, Z. Zhu, Y. Liu, J. Chen, T. Chen, P. Ji, Q. Wan, J. Wang, *Nat Commun* **2021**, 12.

[2] a)W. Huang, S. Cheng, X. Wang, Y. Zhang, L. Chen, L. Zhang, *Adv Funct Mater* **2021**, 31; b)L. Zhou, C. Dai, L. Fan, Y. Jiang, C. Liu, Z. Zhou, P. Guan, Y. Tian, J. Xing, X. Li, Y. Luo, P. Yu, C. Ning, G. Tan, *Adv Funct Mater* **2021**, 31.

[3] J. Tang, K. Xi, H. Chen, L. Wang, D. Li, Y. Xu, T. Xin, L. Wu, Y. Zhou, J. Bian, Z. Cai, H. Yang, L. Deng, Y. Gu, W. Cui, L. Chen, *Adv Funct Mater* **2021**, 31.

[4] J. Wu, Z. Pan, Z. Y. Zhao, M. H. Wang, L. Dong, H. L. Gao, C. Y. Liu, P. Zhou, L. Chen, C. J. Shi, Z. Y. Zhang, C. Yang, S. H. Yu, D. H. Zou, *Adv Mater* **2022**, 34, e2200115.

[5] S. Bai, X. Zhang, X. Lv, M. Zhang, X. Huang, Y. Shi, C. Lu, J. Song, H. Yang, *Adv Funct Mater* **2019**, 30.

[6] Y. Liu, Z. Zhu, X. Pei, X. Zhang, X. Cheng, S. Hu, X. Gao, J. Wang, J. Chen, Q. Wan, *ACS Appl Mater Interfaces* **2020**, 12, 36978.

[7] D. Li, K. Chen, H. Tang, S. Hu, L. Xin, X. Jing, Q. He, S. Wang, J. Song, L. Mei, R. D. Cannon, P. Ji, H. Wang, T. Chen, *Adv Mater* **2022**, 34, e2108430.

[8] a)H. Wei, B. Zhang, M. Lei, Z. Lu, J. Liu, B. Guo, Y. Yu, *ACS Nano* **2022**, 16, 4734; b)Y. Yang, T. Xu, Q. Zhang, Y. Piao, H. P. Bei, X. Zhao, *Small* **2021**, 17, e2006598.

[9] a)Q. He, S. Yuan, H. Tang, S. Wang, Z. Mu, D. Li, S. Wang, X. Jing, S. Hu, P. Ji, T. Chen, *Adv Funct Mater* **2021**, 31; b)Z. Mu, K. Chen, S. Yuan, Y. Li, Y. Huang, C. Wang, Y.

Zhang, W. Liu, W. Luo, P. Liang, X. Li, J. Song, P. Ji, F. Cheng, H. Wang, T. Chen, *Adv Healthc Mater* **2020**, 9, e1901469.
